# Heparan Sulfate Glycosaminoglycans: (Un)Expected Allies in Cancer Clinical Management

**DOI:** 10.3390/biom11020136

**Published:** 2021-01-21

**Authors:** Isabel Faria-Ramos, Juliana Poças, Catarina Marques, João Santos-Antunes, Guilherme Macedo, Celso A. Reis, Ana Magalhães

**Affiliations:** 1 Instituto de Investigação e Inovação em Saúde (i3S), University of Porto, 4200-135 Porto, Portugal; iantunes@ipatimup.pt (I.F.-R.); jpocas@ipatimup.pt (J.P.); cgoncalves@ipatimup.pt (C.M.); joao.claudio.antunes@gmail.com (J.S.-A.); celsor@ipatimup.pt (C.A.R.); 2 Instituto de Patologia e Imunologia Molecular da Universidade do Porto (IPATIMUP), 4200-135 Porto, Portugal; 3 Molecular Biology Department, Instituto de Ciências Biomédicas Abel Salazar (ICBAS), University of Porto, 4050-313 Porto, Portugal; 4 Pathology Department, Faculdade de Medicina, University of Porto, 4200-319 Porto, Portugal; guilhermemacedo59@gmail.com; 5 Gastroenterology Department, Centro Hospitalar S. João, 4200-319 Porto, Portugal

**Keywords:** biomarker, cancer, cancer therapy, extracellular vesicles, glycosaminoglycans, heparan sulfate, proteoglycans

## Abstract

In an era when cancer glycobiology research is exponentially growing, we are witnessing a progressive translation of the major scientific findings to the clinical practice with the overarching aim of improving cancer patients’ management. Many mechanistic cell biology studies have demonstrated that heparan sulfate (HS) glycosaminoglycans are key molecules responsible for several molecular and biochemical processes, impacting extracellular matrix properties and cellular functions. HS can interact with a myriad of different ligands, and therefore, hold a pleiotropic role in regulating the activity of important cellular receptors and downstream signalling pathways. The aberrant expression of HS glycan chains in tumours determines main malignant features, such as cancer cell proliferation, angiogenesis, invasion and metastasis. In this review, we devote particular attention to HS biological activities, its expression profile and modulation in cancer. Moreover, we highlight HS clinical potential to improve both diagnosis and prognosis of cancer, either as HS-based biomarkers or as therapeutic targets.

## 1. Introduction

After decades of knowledge about the cellular signalling pathways mediated by glycoconjugates and the impact of the glycan structural characteristics in defining specific cellular responses, researchers are taking advantage of the multiple features of glycosaminoglycans (GAGs) to develop new tools for improving the clinical management of cancer. GAGs are long linear chains of heterogeneous saccharides, comprising one of the major biomolecules class found in all mammalian cells [1]. GAGs have been extensively studied, and their interactions with growth factors, morphogens, chemokines, extracellular matrix (ECM) proteins and their bioactive fragments, receptors, lipoproteins and pathogens are well described [1,2,3,4,5]. This dynamic network orchestrates several essential functions, from critical steps in embryogenesis and early development to ECM (re)modelling and cell signalling regulation in various physiological and pathological contexts, such as metabolic and neurodegenerative diseases, infections and cancer [6,7]. This review focuses on one particular class of GAGs: Heparan sulfate (HS). HS are anionic polysaccharide chains that assemble as disaccharide building blocks of glucuronic acid (GlcA) linked to *N*-acetyl-glucosamine (GlcNAc) and undergo extensive modification through the action of at least four families of sulfotransferases and one epimerase. HS chains are covalently linked to a core protein to form heparan sulfate proteoglycans (HSPGs), which can be expressed at the cell membrane, released into the ECM [8] or secreted in extracellular vesicles (EVs) [9,10]. The HSPGs are the main mediators of cellular interaction with an enormous number of ligands. Over the last decade, new insights have emerged regarding the mechanisms and the biological significance of those interactions [7,11,12,13], and in this last couple of years, their biomedical potential has been at the forefront in glycobiology translational research [14,15,16]. HS interfere in many steps of tumour progression, such as cancer cell proliferation, immune response escaping, invasion of neighbour tissues and metastasis [7,9,17]. Moreover, the aberrant expression of different HSPGs and of the key enzymes involved in HS biosynthesis and post-synthesis modifications impact cancer cell behaviour [17,18]. The interplay between researchers and clinicians has been key to identify the major needs in the clinical practice, and therefore, propel a better understanding on the potential of HS with the ultimate goal of improving cancer patients’ management. This ladder could not be scaled without the parallel development of powerful analytical equipment and approaches for glycan characterisation [19,20]. These biotechnological advances have contributed to unravel important features regarding the chemical diversity of HS structures, along with the intricate regulation of its biosynthetic pathways.

This review presents the main HS and HSPGs biological functions, from physiological to disease contexts, and summarises the most recent findings on HS as biomarkers and/or as therapeutic targets.

## 2. Glycosaminoglycans as Main Extracellular Matrix and Glycocalyx Building Blocks

### 2.1. Glycosaminoglycans and Proteoglycans Diversity

ECM is a well-organised and dynamic macromolecular complex that provides a three-dimensional scaffold for cells and contributes to tissue homeostasis. Generally, the ECM is composed by varied fibrous proteins, polysaccharides and water. However, its major components, and subsequent structural features, are tissue-specific. Its most common constituents include collagens, glycoproteins, such as laminins and fibronectin, proteoglycans (PGs) and GAGs [21,22]. Besides acting as important ECM building blocks, PGs are also major components of the cellular glycocalyx. This cell’s surface layer includes a vast group of membrane-attached PGs, secreted GAG chains, glycoproteins and glycolipids being associated with cellular functions in homeostasis, as well as to cell responses to injury and disease [1].

**Figure 1:**
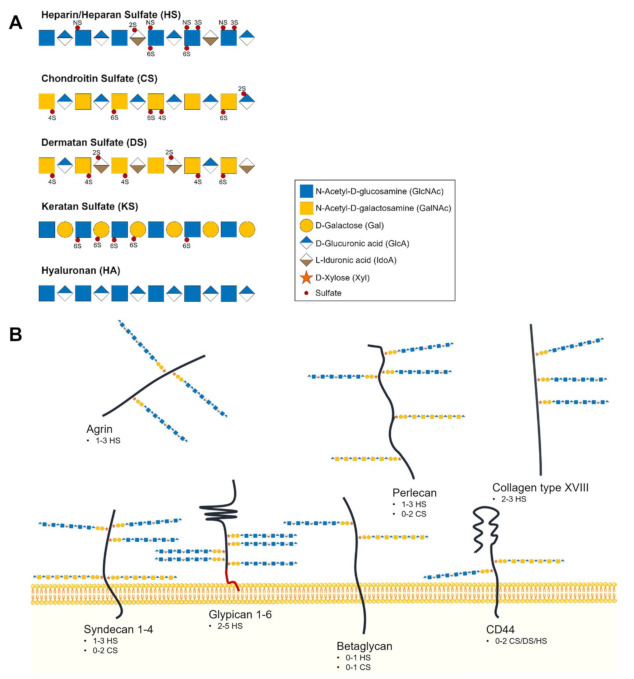
(**A**) Structural composition and classification of glycosaminoglycan chains. Non-reducing termini are to the right of the saccharide’s sequences. (**B**) Illustrative representation of major heparan sulfate (HS)-proteoglycans composing the cells’ glycocalyx and extracellular matrix (ECM). Below each family of heparan sulfate proteoglycans (HSPGs) is indicated the number and type of glycosaminoglycan (GAG) chains that commonly modify the core protein [8,23].

PGs are composed by a core protein with GAG chains covalently attached. GAGs are long and linear polysaccharides composed by repeating disaccharide units and represent an important distinctive structural feature amongst different PGs. According to the disaccharide units that build these chains, GAGs can be classified as HS, chondroitin sulfate (CS), dermatan sulfate (DS) or keratan sulfate (KS) [8]. Hyaluronan (HA) is the exception because it is the only non-sulfated GAG and lacks a covalent bond to a protein core. The different classes of GAGs are schematically represented in Figure 1A.

According to their cellular and subcellular localisation, overall homology and function, PGs can be further classified into five different groups: (i) Intracellular proteoglycans (Serglycin); (ii) Cell surface proteoglycans (syndecans (SDCs), chondroitin sulfate Proteoglycan 4/neuron glia antigen-2 (CSPG4/NG2), betaglycan/TGFβ type III receptor; phosphacan/receptor-type protein tyrosine phosphatase β; glypicans (GPCs)/GPI-anchored proteoglycans); (iii) pericellular and basement membrane proteoglycans (Perlecan; Agrin; Collagens XV and XVIII); (iv) extracellular proteoglycans (Aggrecan; Versican; Neurocan and Brevican); and (v) small leucine-rich proteoglycans (SLRPs) (class I-V), which are abundant ECM glycoconjugates (decorin, biglycan, fibromodulin, luminican, kerotocan, osteoglycan) [24].

The different carriers of HS GAGs at the cellular glycocalyx are shown in Figure 1B.

### 2.2. Heparan Sulfate Biochemical and Structural Features

HSPGs are composed by a core protein with covalently linked HS chains, whose length ranges between 50–400 disaccharide units [25]. The glycan portion directly attached to the protein is termed tetrasaccharide linker and is composed by a residue of xylose (Xyl) directly attached to the core protein, two galactose (Gal) residues and one GlcA residue. This region is followed by repeating disaccharide units of glucosamine and uronic acid residues. It is the sulfation pattern of these repeating units that generate large structural and functional diversity. The glucosamine residues can either be *N*-sulfated (GlcNS) or *N*-acetylated (GlcNAc), both of which can suffer 6-*O*-sulfation (GlcNS(6S) and GlcNAc(6S)). GlcNS and GlcNS (6S) can also be further 3-*O*-sulfated (GlcNS(3S) and GlcNS(3,6S)). The uronic acid residues that can either be GlcA or its epimer Iduronic Acid (IdoA), can also be 2-*O*-sulfated (IdoA(2S) and GlcA(2S)) [6,25,26]. These sulfation and epimerisation reactions give rise to, at least, 23 different HS disaccharide structures that constitute the sulfated (S)-domains subsequently repeated through the chains. The *S*-domains are intercalated by *N*-acetylated (NA)-domains, which are enriched in less modified disaccharides, providing great variability within HS polysaccharides [25].

HS biosynthesis occurs in Golgi apparatus or at the endoplasmic reticulum (ER)-Golgi interface, and is organised in three major events: (i) GAG-protein tetrasaccharide linker assembly, through which HS are covalently attached to particular serine residues in the PG core protein; (ii) HS chains polymerisation; and (iii) structural modifications of the elongated chains [25]. The first two stages include a series of sequential steps catalysed by different glycosyltransferases. It starts with the transfer of a Xyl residue, catalysed by two *O*-xylosyltransferases (XYLT1 and XYLT2), followed by the addition of a Gal residue, by Galactosyltransferase-I/β4-Galactosyltransferase 7 (β4Gal-T7) and subsequent transient phosphorylation of the Xyl residue mediated by the kinase FAM20B. This last step is essential for the following reactions of assembly, as it enhances the activity of subsequent glycosyltransferases, namely, the Galactosyltransferase-II/β3-Galactosyltransferase 6 (β3Gal-T6), which will then add the second residue of Gal to the nascent polysaccharide chain [27,28]. The biosynthesis of the tetrasaccharide linker (GlcAβ1-3Gal-β1-3Gal-β1-4Xyl-β1-*O*-Ser) is completed once the Glucuronyltransferase I (GlcAT-I) adds a GlcA residue to the extremity of the chain, in a reaction step that occurs simultaneously with the dephosphorylation of the Xyl residue by the 2-Phosphoxylose phosphatase (XYLP) [29].

Knock-out (KO) cellular glycoengineering showed that abrogation of XYLT2 in CHO cells that do not express XYLT1, abolished HS biosynthesis. Additionally, elimination of *B4galt7* and *B3gat3* (GlcAT-I) gene expression also fully impaired GAGs biosynthesis, while the KO of the genes coding for the enzymes β3Gal-T6 and FAM20B only reduced its synthesis [30]. Koike et al. conducted silencing experiments in HeLa cells and observed great reduction of the levels of HS chains in lower XYLP expressing cells, suggesting that the dephosphorylation of xylose residues is necessary for correct tetrasaccharide linker assembly [29]. However, more recently, it was determined that the KO of *Pxylp1*, performed on CHO cells, did not alter the levels of GAGs [30]. These results indicate that the role of XYLP in the maturation of the tetrasaccharide linker might be dependent of the cellular context.

The above-mentioned enzymatic steps are common to the biosynthesis of heparin/HS and CS/DS GAG chains, while the following events dictate the biosynthesis of a particular type of GAG chains. Focusing on HS, the addition of a GlcNAc residue to the linkage tetrasaccharide initiates the polymerisation of these chains (in detriment of the polymerisation of CS chains). This reaction involves the catalytic activity of two members of the Exostosin (EXT) family, EXT-like proteins 2 and 3 (EXTL2 and EXTL3), and is followed by further elongation promoted by a hetero-oligomeric complex formed by EXT1 and EXT2 that mediates the intercalated transfer of GlcNAc and GlcA residues [31,32,33,34].

EXTL3 acts as a highly efficient α1,4-GlcNAc transferase towards mature tetrasaccharide linkers by adding the first GlcNAc to the HS chains [32]. Different in vitro and in vivo models have revealed that KO of *EXTL3* results in the abolition of HS biosynthesis, uncovering the crucial role of this enzyme in initiating the elongation of HS chains [30,35,36]. The regulatory activity of EXTL2 in this step stills raises significant doubt. EXTL2 is characterised as an α1,4-*N*-acetylhexosaminyltransferase, displaying dual in vitro catalytic activity by adding both GlcNAc and GalNAc residues to linker mimetics. It has been demonstrated that this glycosyltransferase cannot add GlcNAc residues to mature tetrasaccharide linker substrates [33], however it exhibits significant *N*-acetylglucosamine-transferase activity towards phosphorylated forms of the tetrasaccharide linker. By adding a GlcNAc residue to immature linker structures (GlcAβ1–3Galβ1–3Galβ1-4Xyl(2-*O*-phosphate)-β1-*O*-Ser), EXTL2 promotes the synthesis of phosphorylated pentasaccharides (GlcNAcα1-4GlcUAβ1–3Galβ1–3Galβ1-4Xyl(2-*O*-phosphate)-β1-*O*-Ser) that neither EXT1 nor EXT2 can further polymerise, ultimately resulting in premature HS chains termination [37]. This is in accordance with the increased HS content reported in EXTL2 KO cell models [30] and EXTL2 deficient mice [37,38].

Once polymerised, HS chains are matured by HS modifying enzymes, including *N*-Deacetylase/*N*-Sulfotransferases (NDST1-4), C5-epimerase and different Sulfotransferases (2OST, 6OSTs, 3OSTs) and sulfatases (Sulf-1 and Sulf-2) [6,39]. HS chain features are not directly encoded by the genome, showing a high level of heterogeneity and large structural diversity in terms of monomer sequence, chain length and sulfation profile, all due to post-translational modifications regulated in the Golgi [25]. Therefore, the resulting HS chains are involved in multiple biological processes, varying over different organs [40,41], stages of development [42,43,44] and pathologies [45,46]. HS chains sulfation and length are crucial to the roles displayed by HSPGs, as these determine the binding affinity to the respective targets. The HS sulfation degree, in particular, confers high negative charge to GAGs, prompting HSPGs to interact, in a non-covalent ionic manner, with several proteins [12].

## 3. Heparan Sulfate Biological Activities

### 3.1. In Physiology

**Figure 2:**
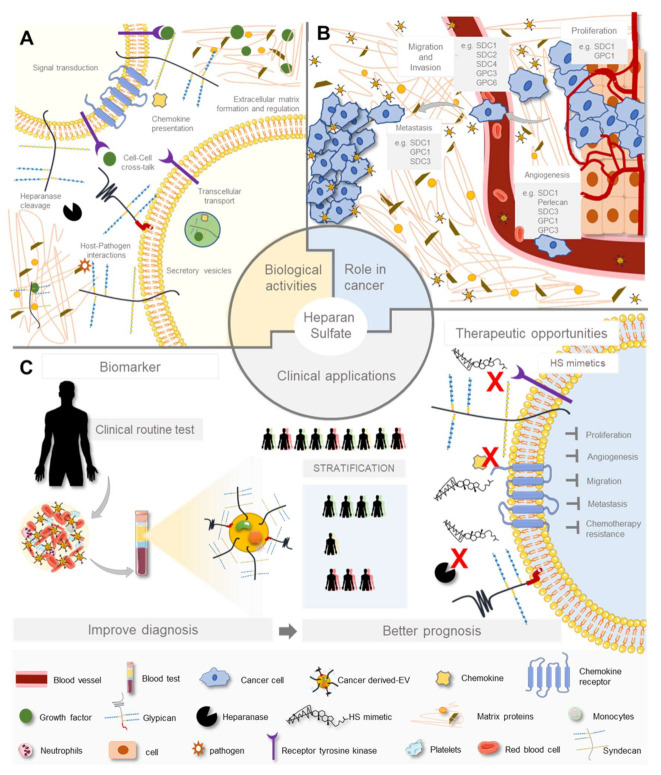
Overview of heparan sulfate proteoglycans functions. (**A**) HSPGs roles in cellular activities, (**B**) HSPGs aberrant expression and functional implications in cancer and (**C**) HSPGs biomedical potential as a biomarker and as a therapeutic target regarding cancer improved diagnosis and prognosis.

HS are loaded with biological roles, as illustrated in Figure 2A. Acting as mediators in a multitude of regulatory mechanisms, ranging from embryonic development to ECM assembly and regulation of cell signalling [6,47,48]. HS interact with a plethora of molecular partners, including soluble proteins (growth factors, morphogens and chemokines), ECM proteins, bioactive fragments and membrane receptors, such as integrins and receptor tyrosine kinase (RTKs). HS chains also promote pathogen attachment and invasion of specific tissues by binding to numerous microorganisms, including viruses, bacteria, parasites and fungi [2,5,49,50,51]. Moreover, HSPGs are expressed in all main organ systems having essential roles in several biological activities like metabolism regulation, transcellular transport, cellular communication, ECM support and modulation. The classical role attributed to cell surface HSPGs was to assist as signalling co-receptor for growth factors activity, allowing a correct presentation to their cognate receptors and helping to stabilise gradients, to control the range of signalling and to protect the proteins against degradation [1]. However, it has been increasingly accepted that besides these co-receptor functions, HSPGs stand alone as key regulators of cell behaviour [52].

During embryonic development, HSPGs modulate the morphogen gradients distribution and other extracellular ligands signalling involved in the formation of the different tissue architectures [53]. In this light, the particular interaction of HS with the Hedgehog signalling pathway is very important to a proper embryonic development [54]. Similarly, HSPGs, being the most abundant PGs in basal lamina and cell surface of skeletal muscle, have been shown to regulate fibroblast growth factor (FGF)**,** Wnt and bone morphogenetic protein pathways, fundamental for the development of skeletal structures [55].

More recently, it was revealed that SDCs can regulate calcium channels of the TRPC (transient receptor potential canonical) family, with functional consequences on the actin cytoskeleton, cell adhesion, junctions and migration. Moreover, this interaction was suggested to be evolutionary conserved and relevant for the progression of some diseases [48,56].

HSPGs are also important modulators of metabolism, as illustrated by their role in the liver mediated clearance of triglyceride-rich lipoproteins [57]. Additionally, several SDCs and GPCs have been implicated in the uptake of different forms of lipoproteins [58,59,60].

Given the many essential cellular and developmental processes in which HS and HSPGs are involved, it is expected that modifications in HSPG expression and structure contribute to a dysregulation in function and lead to pathological scenarios, such as cancer [12,17]. In Section 4, we address several cancer cellular features that are regulated by changes in expression, glycosylation and sulfation profiles of HSPGs, which in turn translate into cancer progression.

### 3.2. In Inflammation

Inflammation represents a first line protection mechanism for any harmful stimuli. Some of the major events during inflammation are regulated by HS, ranging from immune cells recruitment, adhesion and rolling, to transmigration phenomena [61]. Changes in the expression of HPSGs and HS differ depending on the type of inflammatory stimuli [62]. One of the main roles of HS and HSPGs is to drive the extravasation and migration of inflammatory cells from the vasculature into tissues, where they establish and provide cytokine gradients [63]. Moreover, HSPGs are involved in developing the basement membrane barrier, providing a structure for epithelial support and regulating the transport of solutes [61,64,65]. Perlecan, agrin and collagen XVIII in ECM and basement membrane interact with matrix proteins, such as fibronectin and laminin, and provide support, resistance to mechanical stress and filtration barrier properties [66]. Ultimately, HS chains of HSPGs bind to growth factors that are involved in tissue growth and repair, making them available at sites of tissue remodelling [67].

### 3.3. In Host-Pathogen Interaction

HS chains provide the gateway for many microorganisms, ranging from normal microbiota to various pathogenic bacteria, viruses and parasites, by mediating adherence to the host cells. This is a crucial step for infection to occur and pathogens exploit the host HSPGs to accomplish it and invade host cells. The previously described structural diversity of the HSPGs offers multiple binding sites and the degree of variability within tissues results in the tissue-specific tropism of some infectious agents [5,51,68,69].

Among the pathogens that bind to host HS chains are parasites like *Plasmodium falciparum* [70]; bacteria, such as *Escherichia coli* [71], *Pseudomonas aeruginosa* [72], *Borrelia burgdorferi* [73] or *Mycobacterium tuberculosis* [74]; and many viruses, amongst which are found Human Papilloma virus, Herpes viruses and Human Immunodeficiency Virus-1 [75,76]. In addition, recently it was reported that severe acute respiratory syndrome coronavirus 2 (SARS-CoV-2) entry in human cells is mediated through the binding to HS chains in a length and sequence-dependent manner [49,77,78,79,80]. The interference with the HS-mediated adhesion steps can represent an effective therapeutic approach for these pathogens and can be achieved by the competition with HS mimetics and other highly sulfated polysaccharides [81]. In addition, some pathogens can release GAGs from host cell surfaces and ECM, and use these solubilised GAGs to coat their surface, deceiving and eventually escaping immune detection [82].

## 4. Heparan Sulfate and Heparan Sulfate Proteoglycans Aberrant Expression in Cancer

### 4.1. Heparan Sulfate Changes in Cancer

Several hallmarks of cancer, such as continuous growth signalling, abrogation of apoptosis, deregulated metabolism, immune evasion and angiogenesis are boosted through pathological alterations of normal physiological processes [83,84,85]. There is cumulative evidence that changes in cellular glycosylation are concomitant with the acquisition of cellular features involved in tumour growth and progression and ECM remodelling. The glycosylation alterations described in cancer include the expression of truncated *O*-glycan structures, increased expression of branched *N*-glycans, de novo expression of sialylated glycans, altered fucosylation and aberrant PGs expression and modification [86,87].

HS chains are key modulators of cancer cell proliferation events, intervening in altered signalling by interacting with growth factor receptors, promoting their dimerisation and consequent activation, leading to overstimulation of downstream signalling cascades [88]. As an example, in multiple myeloma cells, SDC1 was shown to interact with HGF via HS chains, promoting enhanced activation of Met and consequent activation of the PI3K/protein kinase B and RAS-Raf MAPK pathways, which are related to cell proliferation and survival [89]. In addition, the activation of the Wnt/β-catenin cascade in multiple myeloma is also promoted by HS chains of SDC1, leading to cancer cells proliferation [90].

Besides the altered expression of HSPGs, the abnormal activity of HS biosynthetic and post-synthetic enzymatic machinery, which determines HS chains’ length, epimerisation, acetylation and sulfation patterns, is also known as a major event behind HS deregulation in cancer [91]. Comparative studies demonstrated that the expression of genes coding for HS biosynthetic machinery is deregulated in several types of cancer, weighing on its role in carcinogenic events [92]. In colorectal cancer, it was shown the aberrant expression of enzymes that catalyse uronic acid structural changes (epimerisation and 2-*O* sulfation), and enzymes that impact glucosamine residues sulfation pattern (NDST1 and 2, 6OST isoforms 3B, 5 and 6), depending on the anatomical location and the metastatic nature of the tumours [93,94]. HS modifying enzymes were also shown to present deregulated expression on breast cancer tissue samples [46]. As for glycoenzymes that intervene in HS polymerisation, the analysis performed using estrogen receptor positive and triple negative breast cancer cell lines revealed altered expression of EXT1, EXT2, EXTL2 and EXTL3 [95]. HS 6-*O*-sulfation levels, determined by the expression of 6OSTs, have been reported as critical for the activation of epidermal growth factor receptor (EGFR) by heparin biding-EGF (HBEGF), and the consequent increase in the expression of angiogenic cytokines on ovarian tumour cells [96]. In lung cancer, *3OST2* hypermethylation and consequent deregulation, was associated with lung tumourigenesis and poor overall patient survival, possibly resulting from the altered HSPGs ability to interact with proteins participating in cell growth and adhesion [97]. Likewise, the hypermethylation in HS 6-*O*-endosulfatase *Sulf-1* promoter region downregulates its expression in gastric cancer cell lines and tissue samples [98], and the decreased levels of HS 6-O-endosulfatase associate with gastric cancer progression [99]. Conversely, the sulfatase Sulf-2 is described to be overexpressed in hepatocellular carcinomas and associated with worse prognosis [100].

Heparanase (HPSE), a β-D-endoglucuronidase, is the only mammalian enzyme known to cleave HS and is one of the most studied glycosylation-related enzymes in cancer [101,102]. This enzyme is known to be a tumour inducer acting in several signalling pathways modulating angiogenesis, cell proliferation, migration and metastasis [103,104,105]. HER2- and EGFR-positive breast cancer cells resistant to lapatinib, a tyrosine kinase inhibitor that blocks the activation of the EGFR and HER2 pathways, revealed increased activity of HPSE. This feature was associated with enhanced activation of EGFR, FAK and ERK1/2 signalling pathways and subsequent cell growth. HPSE inhibition, was shown to sensitise these cells to lapatinib and inhibit formation of brain metastases [106]. More recently, vascular endothelial growth factor receptor 3 (VEGFR3)-expressing macrophages and cathepsin release, both playing a significant role in metastasis formation in chemotherapy-treated tumours, were found to promote lymph angiogenesis as a result of VEGF-C upregulation by HPSE [107]. Autophagy is another cellular attribute modulated by HPSE. This catabolic pathway maintains homeostasis in normal cells, while it is completely hijacked in several tumours, promoting cancer cell survival. Autophagy induced by lysosomal HPSE has been shown to enhance tumour development and chemoresistance [108,109,110]. Although HPSE activity has been mainly described extracellularly or within the cytoplasm, nuclear HPSE has also been reported [111,112,113]. In melanoma, nuclear HPSE was shown to suppress tumour progression by competing for DNA binding and inhibiting the transcription of genes, such as those coding for ECM-degrading enzymes that promote metastasis formation [112]. In multiple myeloma disease context, HSPE was recently associated with chromatin opening and transcriptional activity concomitant with downregulation of PTEN tumour suppressor activity [111]. Also supporting the role of HPSE in tumour progression and metastasis formation, HPSE has been shown to promote EV secretion by tumour cells, affecting its protein cargo [114,115], and modulating HS structure on recipient cells to facilitate EVs internalisation [9,115,116].

### 4.2. Role of Heparan Sulfate and Heparan Sulfate Proteoglycans in Cancer Cellular Features and Extracellular Matrix Remodelling

Cancer cells undergo relevant morphological changes, such as the epithelial to mesenchymal transition (EMT), to increase motility capacity. HS chains play a key role in this transition, due to their binding affinity to key growth factors secreted into the tumour microenvironment [117,118]. Particularly, upregulation of SDC4, in lung adenocarcinoma, was shown to stimulate cell’s migration and invasion via TGF-β1, accompanied by induction of EMT [119]. Cell proliferation is also a crucial characteristic of malignant transformation. The HSPGs GPC1 and SDC1, overexpressed in a high percentage of breast cancer pathologies, enhance the mitogen effects associated with heparin-binding growth factors like Basic Fibroblast Growth Factor (FGF2), HBEGF and Hepatocyte growth factor (HGF), promoting cell proliferation [120]. Some studies have shown that HS and HSPGs can translocate to the nucleus and contribute to gene expression regulation [121,122]. Although the role of nuclear HSPGs is still not fully uncovered, another important role of nuclear HSPGs is the translocation of specific cargo to the nucleus. Nuclear translocation of SDC1 was shown to regulate tumour signalling by shuttling growth factors to the nucleus and by altering histone acetylation [123].

Tumour progression is accompanied by the development of new blood vessels [124]. HS chains, by binding to angiogenic growth factors, namely, FGFs, platelet-derived growth factors and VEGFs, dictate HSPGs relevant roles in angiogenesis [91,125,126]. It has been shown that HS presence on endothelial-cells’ surface can serve as a binding site for the potent antiangiogenic factor endostatin. Several studies have indicated that the HS binding site for endostatin is distinct from that of pro-angiogenic factors, such as FGF. This raises the possibility that endothelial cells modulate their HS cell-surface profile to become either more or less sensitive to angiogenic signals from a growing tumour [127]. As referred earlier, SDC1 overexpression in multiple myeloma was also shown to promote angiogenesis by its ability to physically interact with VEGFR2 and prevent the receptor recycling [128]. Perlecan is also an important player in angiogenesis, since its expression is abnormally high in the basement membranes of highly metastatic human melanoma tumour cells [129], promoting the binding of pro-angiogenic FGF2 to its receptors, and consequently increasing angiogenesis [130]. In addition, SDC3 expression is positively associated with angiogenesis in neuronal and brain tissues [131,132].

Beyond the structural modifications of HS and pattern of sulfation, mentioned in Section 4.1, alterations in the HS levels can compromise the stiffness of the ECM, thus modulating cell adhesion and migration. A steady ECM does not offer the best conditions for cell migration, preventing or delaying cell motility. In this light, the ability of cancer cells to invade the surrounding tissues is modulated by changes in the expression of HS and HSPGs, which mediate several events of cell-matrix interaction, and the secretion of HPSE and metalloproteinases that allow cells to penetrate the basement membrane and ECM to invade surrounding tissues [118,133,134]. In hepatocellular carcinoma, SDC1 and SDC4 are key for migration, invasion and increased motility mediated by chemokine-SDC interactions [135]. SDC1 abnormal expression, for example, is determinant in tumour cell growth, invasion and migration in different types of cancer, such as colorectal, gallbladder and oesophageal carcinomas [136,137,138,139]. SDC2 overexpression in breast [133,140], colon [141] and pancreatic [142] tumour cells, is associated with altered cell morphology, focal adhesion formation, spreading, enhanced migration and invasion capabilities, and overall to a more aggressive tumour cell behaviour and disease progression [143]. GPCs are also frequently reported to play a part in cancer progression. GPC1, for instance, when upregulated, increases tumour angiogenesis and metastasis in pancreatic cancer [144,145]. In addition, GPC1 modulates heparin-binding growth factors and plays a role in tumour progression in breast cancer [46,120]. In esophageal squamous cell carcinoma and glioblastomas, GPC1 is also upregulated and associated with tumour angiogenesis and patients’ poor prognosis [146,147,148]. GPC3 is overexpressed in hepatocellular carcinomas tissues [149], and associates with higher invasion and migration [150]. Similar to GPC1, an increased expression of GPC6 has been reported in breast cancer. GPC6 overexpression stimulates cancer invasion through NFAT (nuclear factor of activated T-cells) signalling pathway–previously reported as an inducer of pro-invasion and migration gene expression [151].

Cancer cells ability to penetrate blood vessels is preponderant for metastatic spread and is followed by circulation through the intravascular stream and establishment in other sites [152]. During the process of metastasis formation, the reorganisation of HSPGs in the ECM, creates an opportunity for new partners to bind to tumour stroma. This process also involves interactions between cancer cells and platelets, endothelial cells and host organ cells, being HS implicated in the formation of tumour metastasis in sites, such as the liver, lungs or spleen [91,153]. Moreover, SDC1 expression was shown to decrease in hepatocellular [154] and colorectal [155] carcinomas, resulting in more invasive phenotypes, with higher metastatic potential.

HS can also contribute for the immune system deceiving to either disregard or promote the tumour growth [156]. In breast cancer for example, SDC1 has been suggested to act both as a regulator of cancer stem cell (CSC) phenotype and as a modulator of lymphocytes, in particular of T helper cells, depending on the subtype of the disease [157].

A schematic representation of HSPG functional implications in cancer progression and their clinical potential is illustrated in Figure 2B,C.

### 4.3. Heparan Sulfate Roles in Cancer Intercellular Communication

Cancer cells communication within the tumour microenvironment is key to defeat stromal challenges, settle and colonise distant sites, leading to metastasis. Despite being the main cause of cancer therapy failure and responsible for the greatest number of cancer-related deaths, metastasis remains poorly understood [158]. It is widely recognised that the process of cancer cell systemic circulation and the development of metastasis requires the participation of several glycoconjugates [86,159].

For years, EVs were thought to be a reservoir of cells undesired material. However, in the last two decades, they have emerged as main players in cellular communication [160,161]. EVs are delimited by a lipid-bilayer and can be classified into different classes, including exosomes, microvesicles and apoptotic bodies. Briefly, EVs are secreted to the environment by all cells and carry bioactive cargo that deliver signals and induce several pathophysiological events in the ECM and recipient cells [162,163]. Regarding cancer, EVs have been demonstrated as important signalling nanoparticles in pre-metastatic niche definition and metastasis [164,165].

The EV cargo includes nucleic acids, proteins, lipids and metabolites [166]. Although several seminal studies have addressed in detail the lipid, protein and nucleic acid contents of EVs, the glycans, and particularly GAGs, remain poorly characterised. However, their biological importance is emerging [160,167,168].

**Figure 3:**
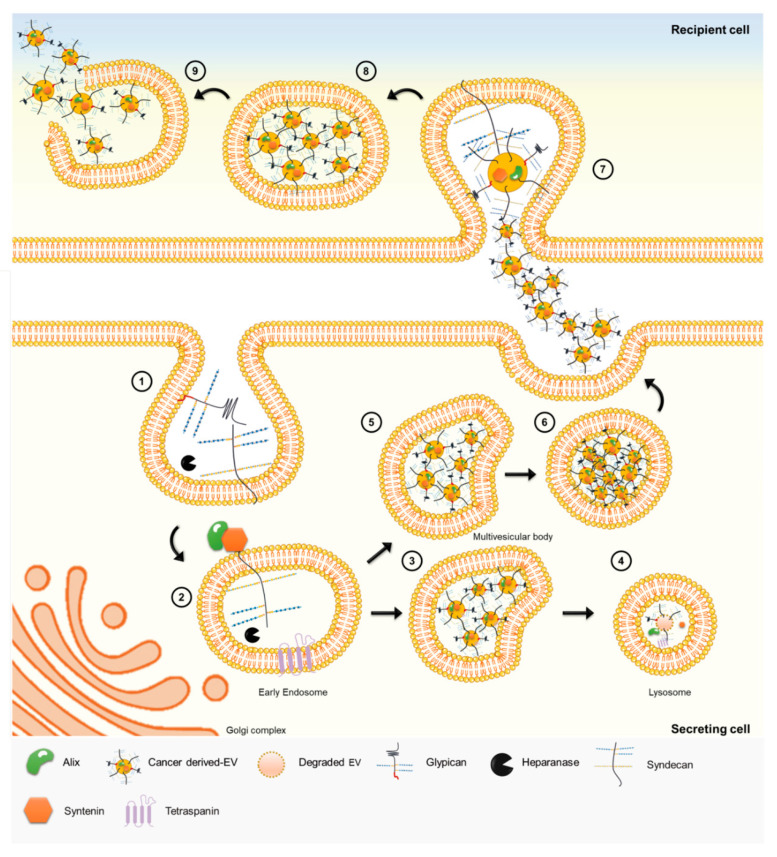
Heparan sulfate proteoglycans regulate EV biogenesis and uptake. (**1**) Cell surface HSPGs can bind multiple ligands through their GAG chains [9]. GAGs can be modified by heparanase activity [174]. (**2**) Syndecan is internalised through endocytosis process, leaving the cytosolic domain clear for syntenin and Alix proteins binding [160,171]. The early endosomes generate the MVBs by inward budding of theirmembrane. (**3**) EVs, particularly those enriched in tetraspanin-6 (TSPN6), can end on lysosome [10] (**4**) with consequently degradation of their content. (**5**) Alternatively, EVs generated inside of the MVBs can be expelled from the secreting cell, through exocytosis. (**6**) After fusion with cellular membrane EVs are released to the extracellular milieu. (**7**) HSPGs, and specifically GAGs, are important receptors of the cell membrane-EV surface cluster and are directly involved in EV uptake by recipient cell [116]. (**8**) After, the complex is internalised by the recipient cell. (**9**) EV-endosome membrane fusion occurs, and EV content is released to cytoplasmic compartment of the recipient cell, and new biological information is transferred [162].

Importantly, HSPGs have been described as key regulators of EVs biogenesis (Figure 3) [162,169]. The biogenesis of EVs depends on the small intracellular adaptor syntenin [170], its interaction with SDC [169] and the endosomal-sorting complex required for transport accessory component ALIX [171,172,173]. Moreover, HPSE can stimulate intraluminal budding of SDC-syntenin-ALIX complex promoting EVs secretion [174,175,176]. Recently, it was described that tetraspanin-6 (TSPN6) may act as a negative regulator of exosomes release through the promotion of SDC4 and syntenin degradation. This interaction highlights the importance of the interplay between these membrane glycoproteins to produce exosomes [10].

Furthermore, it has been demonstrated that HSPGs, namely, SDCs and GPCs, are critical internalising receptors of cancer cell-derived EVs and determine their functional activity (Figure 3) [116,160]. Very recently, it was shown that under hypoxia stress, the uptake of EVs is upregulated, through a mechanism dependent on HSPG receptors and lipid raft mediated endocytosis [177].

EVs exhibit several distinctive features, from a longer half-life provided by increased resistance to degradation, therefore offering their cargo a higher stability, to the ability of travelling long distances. Furthermore, EVs can carry multiple cargo possibilities and also exhibit a unique interactive surface area [178], which may establish contact with both cells and components in the ECM microenvironment [179]. HSPGs are herein important mediators with several functions from EV secretion and trafficking to their uptake [60,116,180].

Taking together glycan and EVs functional relevance in cancer development, it is not surprising that glycans in EVs have been implicated in cancer cells proliferation, angiogenesis, therapeutic resistance [181], control of metabolic activity, and immune system evasion mechanisms [160].

### 4.4. Impact of Heparan Sulfate in Cancer Cell Resistance to Therapy

Cancer treatment relies upon four main approaches: surgery, radiation therapy, chemotherapy and immunotherapy. Some individuals will only require one treatment, but most often, a combination of treatments is used to tackle the resistant nature of cancer. Surgery can be used for solid tumours that are located in reachable areas of the body. Nevertheless, many cancers are metastatic or have a high risk for metastasis formation, implying the use of more aggressive treatments, such as radiotherapy and chemotherapy [182].

A great body of evidence indicates that tumour sensitivity to drug treatment is affected by glycosylation, particularly by altered expression of cell-surface HSPGs and/or HPSE [17,87]. GPCs and SDCs are usually implicated in chemo-resistance. GPC3 overexpression has been described to lead to a decrease in the accumulation of drugs associated with atypical multidrug resistance in gastric cancer [183], and high levels of GPC1 expression in patients with oesophageal squamous cell carcinoma are related to chemo-resistance [146]. As previously mentioned, SDC1 overexpression correlates with a malignant phenotype and, in addition, it is also implicated in resistance to cytotoxic or targeted therapeutics in breast cancer and multiple myeloma [157,184,185,186,187,188]. SDC1 levels in pre-chemotherapy breast cancer biopsies correlate with decreased response to treatment with cyclophosphamide and epirubicin [189]. Additionally, the sensitivity of breast cancer cells to trastuzumab is associated with the availability of HS chains on the cell surface and their ability to elicit the antibody response by forming a ternary complex with trastuzumab and HER2 [190].

Cancer cells resistance occurs not only to chemo, but also to radiotherapy. For example, in pancreatic cancer, HPSE was found to be overexpressed throughout the process of ionising radiotherapy, resulting from the downregulation of the transcription factor EGR1, which leads to the upregulation of HPSE and promotes tumour cells invasion [191]. In cervical cancer, HPSE expression was shown to enhance angiogenesis and radiation resistance through the hypoxia-inducible factor 1 (HIF1) pathway [192]. In addition, the interplay between SDC1 and HPSE, through indirect stimulation of HSPG shedding by metalloproteinases, and consequent activation of HS-binding growth factor signalling, was suggested to associate with colorectal cancer cells resistance to chemotherapy [193]. Clinical drugs used for myeloma showed to induce SDC1 shedding, due to the upregulation of HPSE expression. Then, HPSE can be internalised by both tumour cells and macrophages, promoting the transcription of pro-tumourigenic genes. This paradox effect leads to tumour recurrence by recreating a new cancer microenvironment, which induces chemo-resistance [188,194]. On the other hand, as referred to in Section 4.1, HPSE can modulate EVs cargo and enhance its secretion. In breast cancer models, it was shown that in a chemotherapy context, the production of EVs is increased, with upregulation of the levels of annexin A6 and promotion of metastasis [195].

It is important to note that several enzymes are involved in GAGs remodelling, and alterations on their activity can lead to the activation of compensatory routes. The variable levels of HS, CS/DS and HA over each other can lead to unusual GAG profiles, which need to be considered when a GAG-target therapeutic approach is being evaluated, since these biomolecules compete for some common substrates. [196].

## 5. Heparan Sulfate Clinical Applications–Recent Advances

### 5.1. Adding Heparan Sulfate to the Equation

The knowledge of the molecular mechanism underlying cancer biology has mightily increased over the last 30 years. The current challenge is to translate this information into benefits for patient care and to convert the new molecular information into therapy and better non-invasive biomarkers.

One of the most obvious cancer-related challenges is the heterogeneity in cancer biology [197]. Again, the challenge is the molecular and cellular heterogeneity of a single tumour, and among tumours from different patients, resulting in the very difficult task to design and select effective therapies without promoting treatment resistance [198,199]. Hereupon, targeting HSPGs and enzymes involved in HS chain editing, as a new anticancer strategy, seems to be a sweet spot of opportunity to deal with these challenges (Figure 2C).

Several different therapies using GAG-based strategies have been reported, and a few HS-specific treatments are on clinical trials [200]. Many new approaches to cancer treatment are emerging, and targeting HS constitutes a novel promising strategy for cancer clinical management [102,201]. These strategies include inhibition of tumour invasion and metastasis using non-anti-coagulant low-molecular-weight heparin (LMWH) analogues and inhibition of tumour progression using HS-mimetics. In animal cancer models, LMWHs were shown to inhibit metastases, diminish primary tumours and increase survival. However, pre-clinical studies have conflicting findings showing an absence of efficacy in reducing disease progression [202].

Likewise, the HPSE contribution to immune regulation is raising clinical interest. Among HS modifying enzymes, HPSE is definitely one of the most investigated as a cancer drug target [105,175,185,188,203,204]. Early observations of the powerful HPSE inhibitory activity of heparin lead the way for the screening of heparin/HS mimetics as HPSE inhibitors [102,205,206,207,208].

Noteworthy, substantial research has been devoted to elucidating the roles that EVs play in the regulation of both normal and pathological processes, and recently multiple studies have demonstrated their potential as a source of cancer biomarkers referred to as “liquid biopsies” [160,209]. Biomarkers for early detection of cancer are essential to improve patients’ clinical management. Based on several reports showing proof-of-concept results for EV-associated pancreatic cancer biomarkers, GPC1 was the first candidate to enter clinical trials, aiming to evaluate its performance as a biomarker [210]. Consequently, various EV-associated biomarkers have been reported for early detection, diagnosis, treatment monitoring, metastasis burden, prediction and prognosis in cancer patients [211].

Along the next sections, we present some representative examples of HS-related mimetics, therapeutic targets, and biomarkers recently studied, under clinical trials evaluation or already introduced in medical practice.

### 5.2. Heparan Sulfate-Based Therapeutic Opportunities

Based on promising preclinical data, some HS-based therapies are currently under clinical investigation although none was yet approved [212,213,214]. Difficulties in interpreting data with HS mimetics are mainly due to their pleiotropic effects [160,206,207].

Currently, leukocyte-based anticancer therapies like Chimeric Antigen Receptor-T (CAR-T) cell therapy, dendritic cell vaccines and viral-therapeutic delivery exploiting HPSE are being developed [102,215]. HPSE is able to change the function of HSPGs in the tumour microenvironment and is a regulator of several cancer hallmarks [216], namely, angiogenesis and the development of metastasis [105,217]. So, targeting HPSE and modulating its activity is a very valuable strategy to overcome several cancer features and improve disease outcome [102,218].

Another possible opportunity for intervention could be the use of the endostatin domain of collagen XVIII. Endostatin exerts an efficient inhibitory effect on tumour angiogenesis and growth [219,220]. Gastric cancer patients with subsequent liver metastasis are extensively studied, and several phase II clinical trials are using recombinant endostatin (either alone or combined with other drugs) as an anti-tumour agent [221,222,223]. In addition, the effect of endostatin in advanced well-differentiated pancreatic neuroendocrine tumours is being tested [224].

Synthetic peptides that interfere with the interaction between HS and its binding partners, also represent an appealing alternative [225]. This approach allows a specific blocking of interaction with the growth factors, acting on particular pathways. Furthermore, a large number of small molecules can also regulate glycosylation by modulating glycosyltransferases and glycosidases activity [218,226] and are emerging as new therapeutic strategies [226,227,228].

Recently, another class of enzymes have been capturing attention: the 3-*O*-sulfotransferases (3OSTs). These enzymes produce a rare HS modification, glucosaminyl-3-*O* sulfation, which affects the selective binding of several ligands. Interestingly, these enzymes can either act as pro or anti-tumourigenic according to the cellular specific context [229,230]. An important challenge in this field is to determine how the HS glycan sequence and sulfation pattern drive ligand binding specificity.

Furthermore, the development of specific HSPG targeted therapeutic approaches is also being studied. Antibody-targeting of SDC1 has been used alone or combined with chemotherapy to treat multiple myeloma [231], and a clinical trial using CAR-T cells recognising SDC1 suggests that the treatment is safe, well-tolerable and has potential antitumour activity [232]. In the same line, in hepatocellular carcinoma, the strategies of immunotoxin and CAR-T cells against GPC3 are showing promising results and are under clinical trials [233,234,235].

### 5.3. Heparan Sulfate Mimetics Development and Clinical Trials

#### 5.3.1. Heparan Sulfate Mimetics Rational

Heparin derivatives and HS mimetics are drawing great attention for developing new therapeutics for diverse diseases, from inflammation to neurodegenerative disorders and cancer [207]. HS mimetics overcome a problem known for more than half a century in Medicine related to heparin use. Heparin is an anticoagulant drug which has been widely used and remains one of the main drugs for prophylaxis and treatment of thrombosis [236]. Thrombosis is a common complication of cancer patients. The use of heparin has improved the survival rate of cancer patients [237]. The functional roles of heparin seem far more than anticoagulation, since a number of additional beneficial effects have been observed for heparin in other diseases than thrombosis [238]. However, heparin is an animal-derived heterogeneous polysaccharide, therefore the potential risk of contamination and its complicated molecular structure restrict its use. Therefore, and taking in consideration the high structural similarities between heparin and HS, it was tested whether heparin interferes with HS interactions with its ligands mainly through the hamper of angiogenic growth factors, like VEGF and FGF, selectins, and HPSE [239,240]. Indeed, in vitro analyses have shown that heparin is able to inhibit HPSE activity [240]. Since it is important to restrict heparin anticoagulant activity, due to haemorrhagic issues, efforts were made to eliminate the anticoagulation activity by chemical modification.

Roneparstat (SST0001) was the first synthetic product based on this premise. SST0001 is a 100% *N*-acetylated and glycol split heparin synthetic molecule [241]. The SST0001 HPSE inhibitory effect was confirmed on myeloma cell growth, and therefore, it is being evaluated for the treatment of multiple myeloma [242].

The principle of using HS mimetics is to interfere with the interactions between HS and its molecular partners (Figure 2C). There are two types of HS mimetics: (1) Synthetic saccharide-based HS assembled from a backbone sugar structure; and (2) non-sugar scaffold negatively charged with sulfates, sulfonates, carboxylates and/or phosphates [205]. SST0001 and other HS-mimicking compounds main attribution is to inhibit HPSE and compete for HS binding with several growth factors, having impact on cancer by preventing angiogenic and metastatic events [218,243]. In summary, HS mimetics have been shown to enhance antitumour effects, particularly when combined with standard therapies.

#### 5.3.2. Promising Heparan Sulfate Mimicking Molecules

The most promissory HS mimetics that are on clinical trials are: Highly sulfated phosphosulfomannan muparfostat (PI-88) [244], 2,3-*O*-desulfated heparin CX-01 (ODSH) [245], SST0001 [246] and pixatimod (PG545) [214]. The latter was selected from oligosaccharidic HS mimetics of the PG500 series as the best candidate regarding cancer treatment applications [247].

PI-88 is an HPSE inhibitor and also an antagonist of HS-protein interactions. Structurally it is a phospho-mannopentaose obtained through a process of sulfation of a phospho-mannan complex produced by yeasts [243,248]. It was the first one entering clinical trials and, generally, phase I/II studies demonstrated a satisfactory pharmacodynamic profile of this mimetic that was also considered safe and well-tolerated, showing minor anticoagulant effects. As main results, PI-88 has proven to be a suitable candidate as an adjuvant for postsurgical hepatocellular carcinoma in phase II clinical trials [249,250]. Subsequently, it reached Phase III in clinical trials regarding large series of liver cancer patients after hepatectomy. In fact, it is currently waiting for approval to enter routine clinical use [250]. Patients with advanced melanoma were recruited for a Phase I and Phase III clinical trials and PI-88 activity was beneficial [251]. Overall, PI-88 showed encouraging results for melanoma, multiple myeloma, prostate and lung cancer treatment [205].

CX-01 is a low anticoagulant 2-*O*, 3-*O* desulfated heparin derived from porcine intestinal heparin retaining many anti-inflammatory properties. It has been shown to have a particular potential for acute myeloid leukaemia (AML) treatment [245]. This molecule inhibits leukemic stem cells to concentrate on the bone marrow, therefore enhancing chemotherapy treatments. CX-01 specifically binds chemokine platelet factor 4 (PF4), which is responsible for the negative regulation of megakaryopoiesis [252]. Therefore, by interfering with this process, CX-01 is able to diminish chemotherapy-induced thrombocytopenia. Phase I clinical trial on AML patients demonstrated CX-01 to be well-tolerated and more recently, it entered Phase II and showed promising results in combination with the standard chemotherapy treatment [253].

Preclinical models with SST0001 shown the effective inhibition of myeloma growth in vivo [242]. SST0001 progressed to a Phase I open-label clinical trial design to assess the safety and tolerability profile of this compound in patients with multiple myeloma [254]. Recently, SST0001 was advised for Phase II evaluation [254].

The HS-mimicking molecule with the best clinical evaluation so far is PG545 [255]. PG545 is a fully sulfated glucopyranose tetrasaccharide particularly designed with a hydrophobic 3-cholestanyl group [247,255]. The novel characteristic of this molecule is the stimulation of innate immune cell response to tumours. This stimulation is done via activation of natural killer cells [256]. Clinical trials proved PG545 as a satisfactory alternative in patients with advanced solid malignancies in which standard therapies failed [247]. Currently, PG545 is also being investigated as a potential inhibitor of the SARS-CoV-2 [257].

In addition to the role of HS as an emerging class of molecules for therapeutic strategies, HS glycan chains also constitute important cellular markers. These properties make HS important targets for vaccines development strategies [258].

### 5.4. Current Heparan Sulfate-Based Biomarkers Landscape

New biomarkers to improve cancer diagnosis are needed, thus improving patient outcomes. In contrast to RNA, DNA and protein synthesis, HS biosynthesis is not a template-driven process. Instead, as mentioned previously, HS are assembled by the activity of a series of enzymes–turning this biosynthetic pathway into a source of very distinct and specific modifications with diverse applications. The type of HSPG glycosylation was shown to affect the ability of immune cells to infiltrate tumour tissues and engage in the immune response [259]. In addition to the critical roles in multiple aspects of tumour biology, described in the previous sections, HSPGs also have value for clinical diagnosis and prognosis in various cancer types. The alteration of GAG abundances is reflected in body fluids (like blood and/or urine), and the HS levels in plasma can, therefore, predict patient’s prognosis [260]. For all these attributes, HS and HSPGs are promising new biomarkers in cancer since their recognition by other molecules is based in high affinity and exquisite specificity. It is imperative to discriminate among related isomers the specific glycan-binding partner. The development of more sophisticated equipment and techniques have been critical to surpass these limitations. Recently, using Raman micro-spectroscopy it was possible to determine unique and discrete HS profiles of individual live cells, which can be of value for clinical screening purposes. This method can be utilised for identifying specific molecular signatures of HS and HSPGs as markers of cancer [261].

GPCs have been receiving great attention from the research community as promising biomarkers [262]. GPC1 is overexpressed in several cancer types [263,264]. GPC1 can be detected in the urine of prostate cancer patients [265]. Further, GPC1 was shown to associate with the dissemination levels of glioblastoma [148]. The abundance of GPC1 also positively correlates with disease severity of pancreatic cancer patients, independently on surgical treatment, suggesting that GPC1 is a surgery-independent diagnostic biomarker [263,266]. Recently, GPC3 was pointed as a promising candidate for hepatocellular carcinoma diagnosis and immunotherapy, as previously described [267]. Similarly, GPC6 was identified as a putative biomarker for the metastatic progression of cutaneous melanoma, since it is possible to track higher levels of GPC6 in melanoma samples when compared with normal melanocytes [268].

The biological features of EVs, such as long half-life and physical resistance properties, make EVs a unique source of biomarkers [167]. Particularly, the identification of specific PGs in EVs secreted by cancer cells has demonstrated their potential as biomarkers for minimally invasive diagnosis. Two important examples are GPC1 in pancreatic cancer [266] and SDC1 in glioma [269]. Both HSPGs were detected in EVs isolated from patients’ plasma, supporting the concept of a minimally invasive biomarker for patients’ stratification. GPC1 was proven to distinguish healthy individuals and patients with a benign pancreatic disease from patients with early- and late-stage pancreatic cancer. Moreover, GPC1-positive EV levels correlated with tumour burden and the patients’ survival and were shown as a prognostic marker superior to CA 19-9, the serum biomarker currently employed in pancreatic cancer screening [266]. SDC1 was shown to discriminate between high-grade glioblastoma multiforme and low-grade glioma, and therefore, with the potential to improve the management of brain tumour patients that present high risk of surgery-associated complications [269].

The recent development of EV analysis platforms based on microfluidics technologies holds promise for the development of high sensitivity and high-throughput assays of EVs analysis with clinical diagnosis purpose [270].

## 6. Conclusions and Future Challenges

The emerging HSPGs biological functions have largely surpassed their classical role as cellular co-receptors and have highlighted HSPGs as main maestros of cancer cell communication and ECM structuring. The HSPGs cellular interactome is vast and is fine-tuned by the biochemical and structural features of the HS chains. HS lack a template for its biosynthesis, and their structural features result from the dynamic cellular GAGosylation pathways that include the sequential, and in some cases competitive, action of specific enzymes, which may associate to form supramolecular complexes [25]. The structural efforts to produce chemically defined HS oligosaccharides [271] have been crucial for the identification of the molecular determinants of enzymatic activity. However, we are still far from fully understanding the complete regulation of HS structural and functional diversity in health and particularly in cancer.

The recent developments in glycosaminoglycanomics, namely, on the analytical techniques for GAGs profiling in cells and clinical samples (tissues and biological fluids), together with the establishment of computational tools for mining GAG-protein interactions and creation of databases, are contributing to significantly improve the knowledge on the human glycosaminoglycome [19,272]. Moreover, the integration of HS structural features and expression with proteomic and transcriptomic analysis will be crucial for further elucidating HS-ligand interactions and unravel HS structure features associated with specific biological functions [273]. Particularly relevant would be the integration of data on relative abundance and structural features of HS for the definition of cancer-specific profiles. Therefore, it is important to apply the most recent GAG analytical approaches to well characterised clinical samples to identify HS signatures that are cancer-specific. To successfully achieve this aim, it is key to incorporate also knowledge on the dysregulation of HS biosynthetic and post-synthetic modification pathways, as well as on the functional redundancy of different PG core protein families in cancer.

The multidimensional roles of HS and HSPGs in different steps of cancer progression have propelled the development of HS-targeted strategies for cancer diagnosis and treatment [200]. Indeed, in this new period of precision oncology, HS GAGs are currently (un)expectedly emerging as allies to improve cancer clinical management by their potential to detect cancer in early stages, allowing an accurate diagnosis, disease monitoring, patients stratification and improve prognosis.

The HSPGs role in regulating EV release, cargo and uptake is well defined [160], but the implications of altered PG expression and glycosylation features in EV biodistribution and metastasis tropism remain to be discovered. It has become highly relevant to understand the impact of HSPG remodelling, both at the level of the glycan structures and core protein expression, in cancer EV-mediated signalling. Understanding the glycosylation modifications involved in EV-cell interaction and cellular uptake is of major relevance for developing therapeutic approaches targeting EV-HSPG interactions as novel cancer treatment strategies.

## Data Availability

Not applicable.
